# Supplementary figures and images for: Hypericin Inhibit Alpha-Coronavirus Replication by Targeting 3CL Protease
Source: Viruses. 2021 Sep 14;13(9):1825. doi: 10.3390/v13091825 (PMC8473218; doi:10.3390/v13091825)

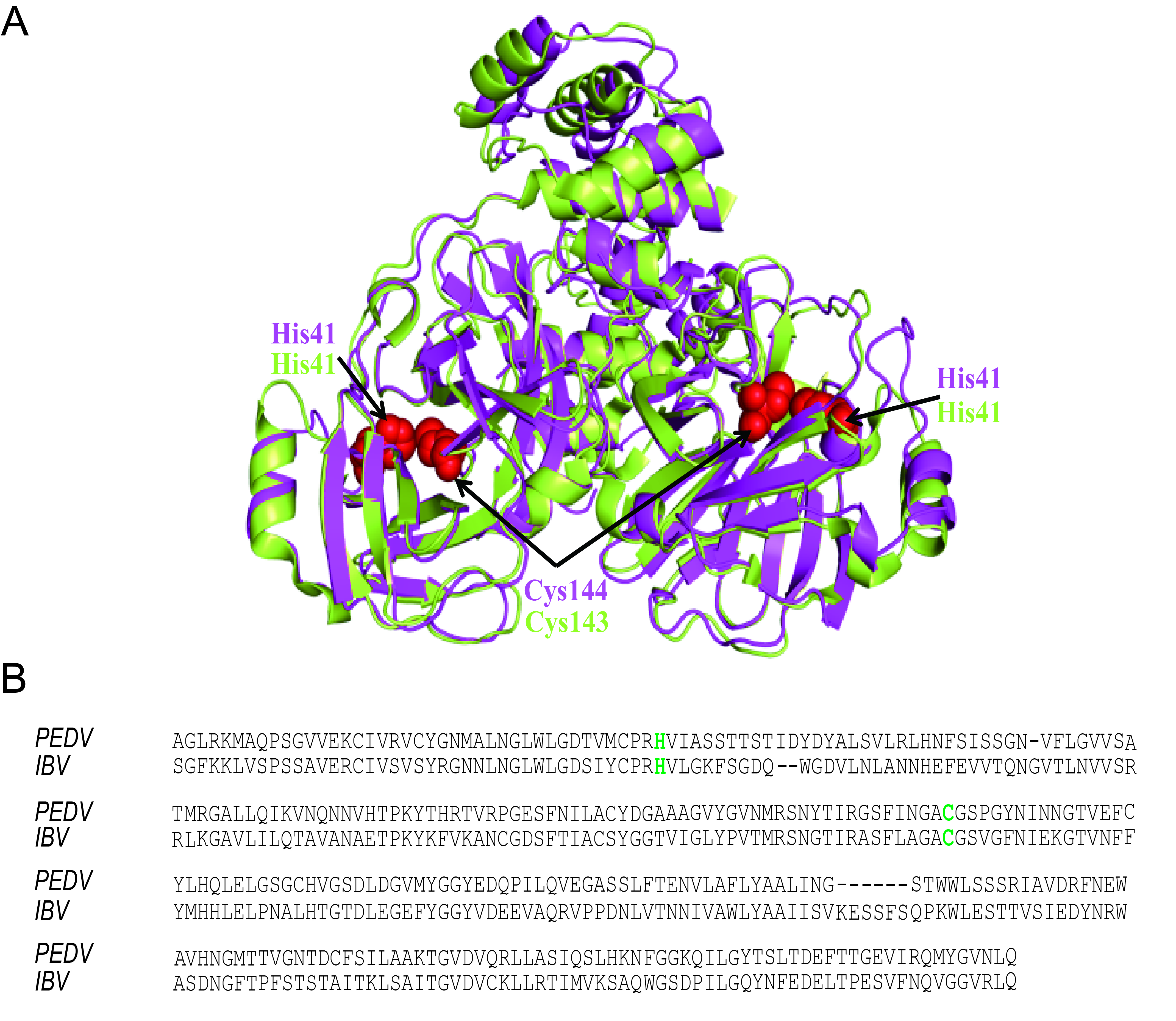

Supplement: Supplementary file 1 [file viruses-13-01825-s001.zip › Figure S1.jpg]

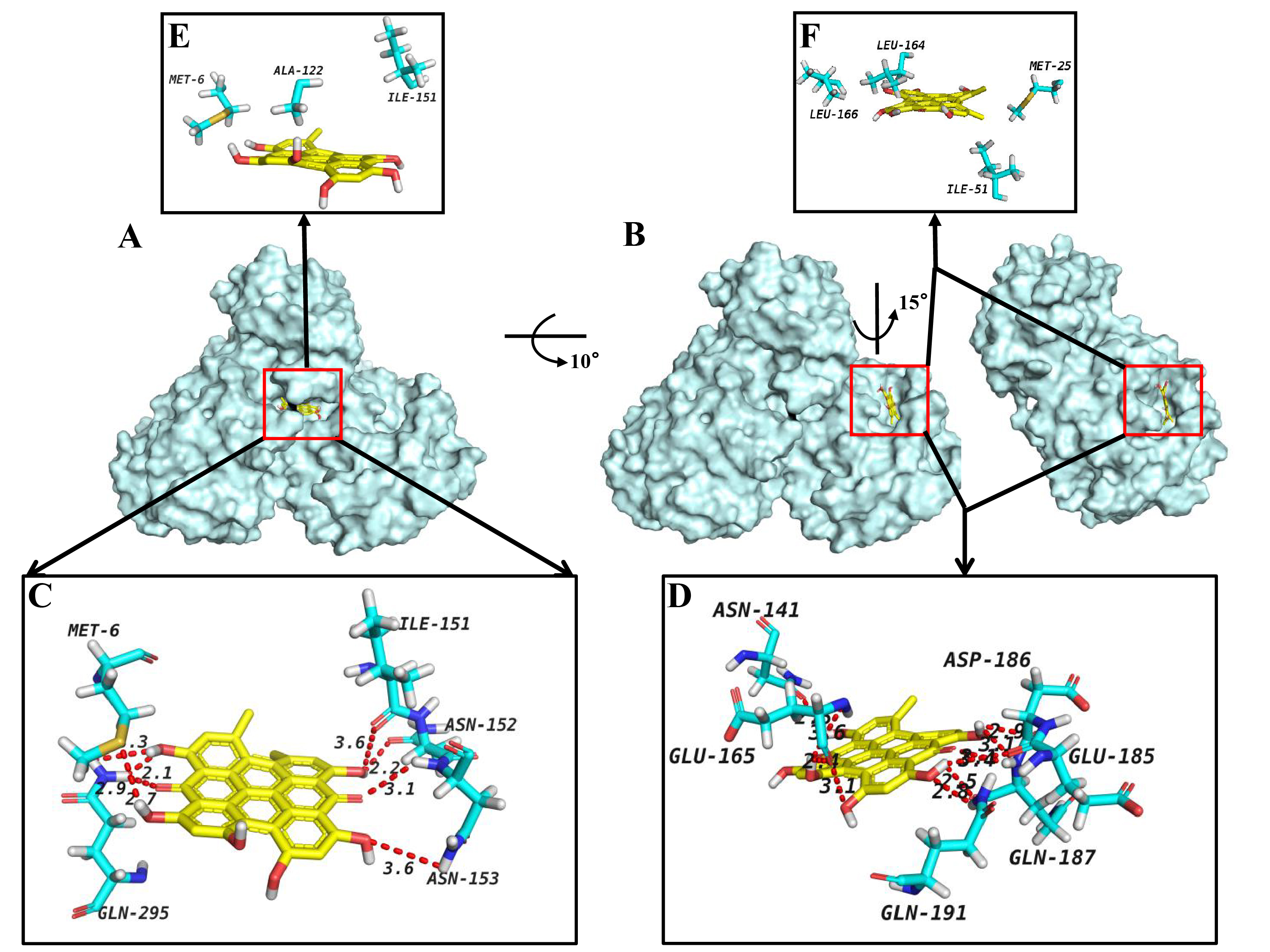

Supplement: Supplementary file 1 [file viruses-13-01825-s001.zip › Figure S2.jpg]

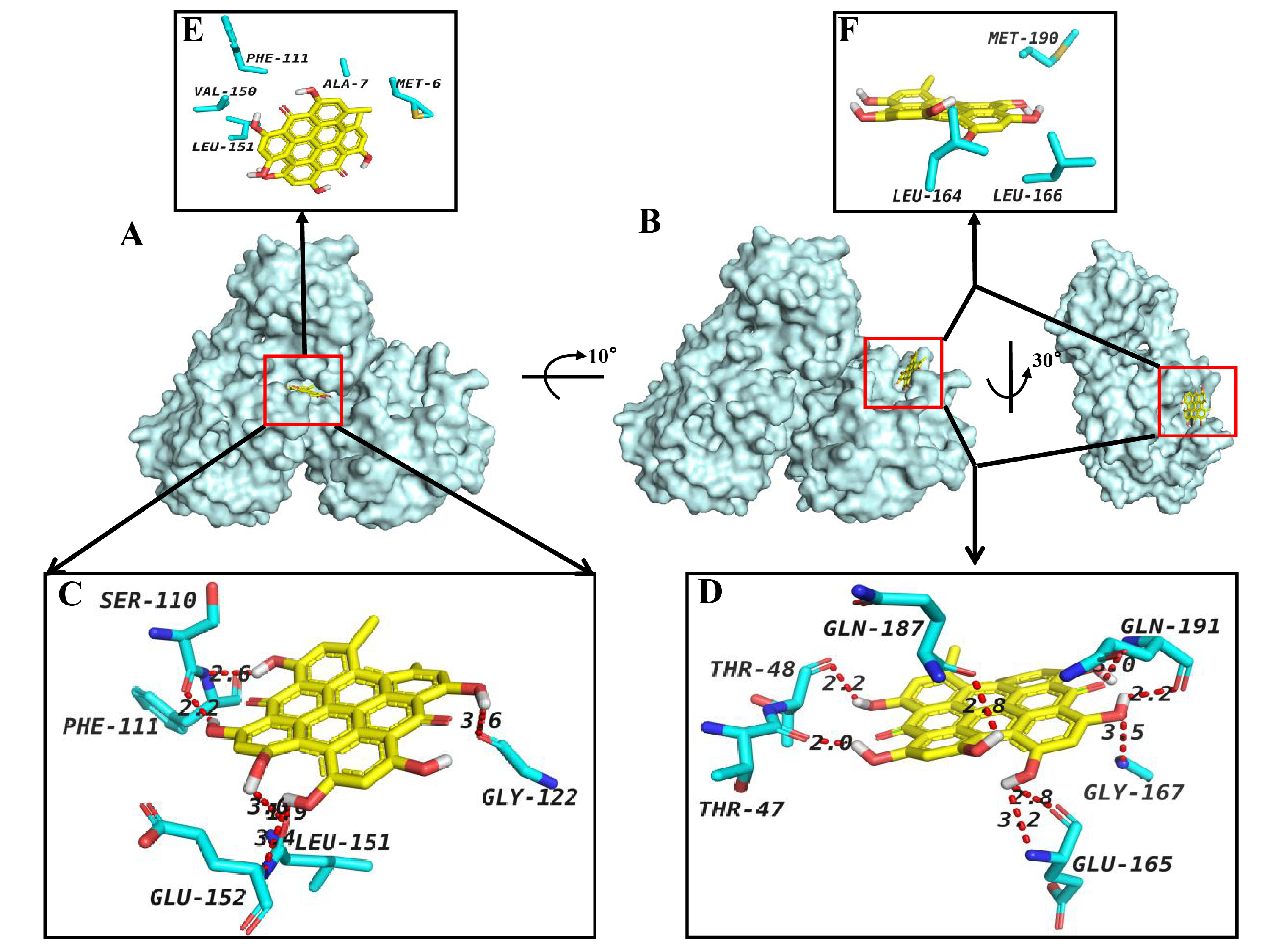

Supplement: Supplementary file 1 [file viruses-13-01825-s001.zip › Figure S3.jpg]

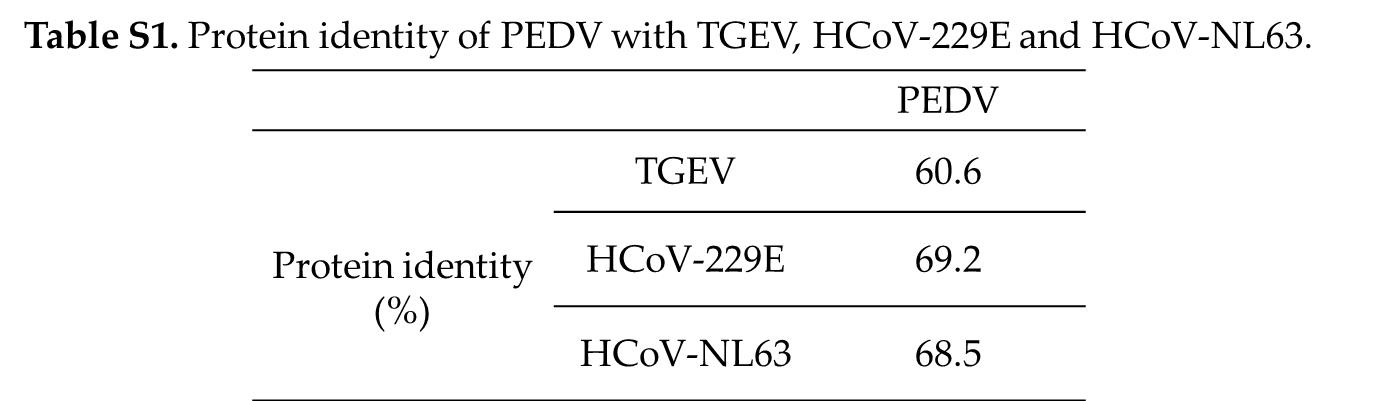

Supplement: Supplementary file 1 [file viruses-13-01825-s001.zip › Table S1.jpg]
